# Supplementary material for: SNooPy: a statistical framework for long-read metagenomic variant calling
Source: Nucleic Acids Res. 2026 Jun 3;54(10):gkag556. doi: 10.1093/nar/gkag556 (PMC13233072; doi:10.1093/nar/gkag556)
Supplement: gkag556_Supplemental_Files [file gkag556_supplemental_files.zip › snoopy_supp_mat_review1.pdf]

Supplementary Material for SNooPy: a statistical framework for long-read metagenomic variant calling

Roland Faure, Ulysse Faure, Tam Truong, Alessandro Derzelle,
Dominique Lavenier, Jean-François Flot, Christopher Quince

April 2, 2026

		strains	coverage	read simulator	reference
Number of strains	2	Y5 H5	20x	NanoSim<br>ONT R10.4.1	Flye assembly
	4	Y5 H5<br>AMSCJX03 EC590	20x	NanoSim<br>ONT R10.4.1	Flye assembly
	6	Y5 H5<br>AMSCJX03 EC590<br>K12 LD27-1	20x	NanoSim<br>ONT R10.4.1	Flye assembly
	8	Y5 H5<br>AMSCJX03 EC590<br>K12 LD27-1<br>ME8067 RM14721	20x	NanoSim<br>ONT R10.4.1	Flye assembly
	10	Y5 H5<br>AMSCJX03 EC590<br>K12 LD27-1<br>ME8067 RM14721<br>SE15 UMN026	20x	NanoSim<br>ONT R10.4.1	Flye assembly
Error rate (%)	0.1	Y5 H5 AMSCJX03 EC590	20x	BadReads<br>ONT2023, 0.1% errors	Flye assembly
	0.5	Y5 H5 AMSCJX03 EC590	20x	BadReads<br>ONT2023, 0.5% errors	Flye assembly
	1	Y5 H5 AMSCJX03 EC590	20x	BadReads<br>ONT2023, 1% errors	Flye assembly
	2	Y5 H5 AMSCJX03 EC590	20x	BadReads<br>ONT2023, 2% errors	Flye assembly
	3	Y5 H5 AMSCJX03 EC590	20x	BadReads<br>ONT2023, 3% errors	Flye assembly
	5	Y5 H5 AMSCJX03 EC590	20x	BadReads<br>ONT2023, 5% errors	Flye assembly
Even coverage	20	Y5	20x	NanoSim<br>ONT R10.4.1	H5
	10	Y5	10x	NanoSim<br>ONT R10.4.1	H5
	5	Y5	5x	NanoSim<br>ONT R10.4.1	H5
	3	Y5	3x	NanoSim<br>ONT R10.4.1	H5
	2	Y5	2x	NanoSim<br>ONT R10.4.1	H5
	1	Y5	1x	NanoSim<br>ONT R10.4.1	H5
Uneven coverage	20	Y5 H5 AMSCJX03 EC590	20x, 20x, 20x, 20x	NanoSim<br>ONT R10.4.1	Flye assembly
	10	Y5 H5 AMSCJX03 EC590	20x, 20x, 20x, 10x	NanoSim<br>ONT R10.4.1	Flye assembly
	5	Y5 H5 AMSCJX03 EC590	20x, 20x, 20x, 5x	NanoSim<br>ONT R10.4.1	Flye assembly
	3	Y5 H5 AMSCJX03 EC590	20x, 20x, 20x, 3x	NanoSim<br>ONT R10.4.1	Flye assembly
	2	Y5 H5 AMSCJX03 EC590	20x, 20x, 20x, 2x	NanoSim<br>ONT R10.4.1	Flye assembly
	1	Y5 H5 AMSCJX03 EC590	20x, 20x, 20x, 1x	NanoSim<br>ONT R10.4.1	Flye assembly

Supplementary Table 1: Description of the experiments run with the simulated datasets on *E. coli*

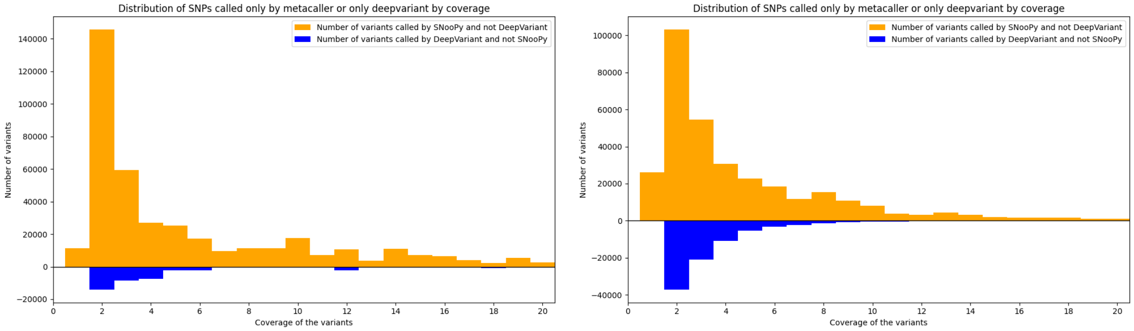

Supplementary Figure 1: Coverage of the variants missed by DeepVariant but not SNooPy, and inversely.
